# Do Body Composition and Values of Selected Nutritional Status Indices Influence the Glycaemic Index Values of Vegetarian Dishes? A Pilot Study in a Group of Older Women

**DOI:** 10.3390/ijerph19169918

**Published:** 2022-08-11

**Authors:** Ewa Raczkowska, Maciej Bienkiewicz, Robert Gajda, Monika Bronkowska, Ewa Piotrowska, Marta Habánová

**Affiliations:** 1 Department of Human Nutrition, Faculty of Biotechnology and Food Science, Wrocław University of Environmental and Life Sciences, 51-630 Wrocław, Poland; 2 Institute of Health Sciences, University of Opole, Katowicka 68, 45-060 Opole, Poland; 3 Institute of Nutrition and Genomics, Faculty of Agrobiology and Food Resources, Slovak University of Agriculture in Nitra, 949 01 Nitra, Slovakia

**Keywords:** glycaemic index, BIA, age, body composition, vegetarian meals

## Abstract

An ageing population brings with it the need for public policy to respond to the demands and health needs of this group of people. The ageing process has been shown to be associated with changes in body composition. These mainly concern a decrease in muscle mass and an increase in body fat. Body composition and other indicators of nutritional status are important factors differentiating carbohydrate management. Glycaemic index (GI) values may be affected by differences resulting from individual metabolism. The rate of carbohydrate digestion is also influenced by a number of factors, including the degree to which the product is processed, the structure of the starch, and the presence of protein, fat and dietary fibre. Available studies do not provide information on the glycaemic response following the consumption of specific products by older people with varying BMI and body composition. Therefore, the aim of this study was to evaluate the effect of the body mass index (BMI) values of women aged 50–80 years on the glycaemic response after eating vegetarian meals and the influence of selected indices of nutritional status on their GI values. It has been shown that the areas under the glycaemic curves after the consumption of the tested foods, both traditional and modified, are higher in the group of overweight and obese women. Nevertheless, the GI of meals consumed by those with a BMI ≥ 25.0 kg/m^2^ is lower than that of foods consumed by women with normal values of this index. In the group of women with BMI 18.5–24.9 kg/m^2^, on the basis of an analysis of the obtained correlations, it was observed that the GI value of modified products depends on the percentage of body fat (FM%) (*p* = 0.0363) and the percentage of fat free mass (FFM%) (*p* = 0.0363), and, in the case of traditional products, also on the percentage of total body water (%) (*p* = 0.0133). In the group of women with a BMI ≥ 25.0 kg/m^2^, significant correlations were only found between the GI of modified foods and the waist-to-hip ratio (WHR) (*p* = 0.0363) and the ratio of waist circumference to height (WHtR) (*p* = 0.0369) indices. The GI values of food set solely with the participation of young, healthy people should not be the basis for the nutrition planning of all groups of people.

## 1. Introduction

According to the World Health Organization, ageing is a biological process that begins at conception and ends at death. This process involves many changes in body composition which are not always reflected in body weight or body mass index (BMI). Our body size is an individual characteristic depending on the age of the individual. It is also influenced by genetic and environmental factors related to lifestyle (physical activity, diet, stress, use of stimulants, interpersonal contacts). There is increasing evidence in the literature showing a correlation between body composition and metabolic health [1,2]. Indicators such as BMI or waist and hip circumferences are often used to assess nutritional status, but they do not provide information about an individual’s metabolic health [3]. In fact, the relationship between body composition and disease risk is a more complex process, depending on the proportion of fat and muscle and their distribution in the body. As we age, the percentage of body fat increases, while the percentage of muscle and bone tissue decreases. Furthermore, the location of body fat in specific areas of the body (android or gynoid obesity) increases the risk of specific diseases. An increase in fat mass (FM), particularly in the abdominal area, may increase the incidence of cardiovascular disease, diabetes or other metabolic diseases. It is generally accepted that a change in body composition is a consequence of an abnormal energy balance. An excess of energy supplied to the body leads to weight gain, whereas too little energy supply leads to weight reduction. However, it should be borne in mind that, among the elderly, these changes often occur in the absence of weight fluctuations [4,5,6].

As we grow older, the diet and the body’s need for nutrients, vitamins and minerals also change. This is due to a number of changes associated with ageing. Some people experience a reduced intake of food, which may be a consequence of a loss of appetite, taste, smell, dental problems or the occurrence of chronic diseases. In addition, the absorption of nutrients from food is also reduced. The living environment is also important and can further exacerbated nutritional deficiencies [4].

Age and body weight are also important factors differentiating carbohydrate management. It is commonly believed that glucose tolerance decreases with age. Studies by Basu et al. have shown that age and gender affect insulin secretion, resulting in significant differences in the regulation of postprandial glucose metabolism in men and women and in older and younger individuals [7,8]. Higher glucose uptake per kilogram of muscle mass was observed in older women compared to that in young women [7]. Yates and Laing found a positive correlation between fasting glucose concentration and age in women; however, this correlation was not significant in men [9]. Among individuals with normal fasting glucose levels, impaired glucose tolerance occurs mainly in elderly, obese or sedentary individuals [10]. The likely reason for this is the increased percentage of body fat in these individuals. Research by Hrubeniuk et al. has proven that physical effort improves glucose tolerance [11]. Its decrease with age results from reduced motor activity; however, impaired insulin secretion is actually the result of the ageing process itself [12]. Moreover, the use of aerobic exercise in the elderly has a beneficial effect on the reduction in abdominal adipose tissue, contributing to lower the fasting glucose concentration and improved sensitivity of tissues to insulin [13,14].

Research conducted by DeNino et al. [15] proved that the content of visceral and subcutaneous fatty tissues increased with age. The experiment was attended by 178 women without obesity, who were classified into four age groups (group I—28 ± 4 years; group II—46 ± 2 years; group III—53 ± 2 years; and group IV—67 ± 6 years). Within the subcutaneous adipose tissue, surface and deep tissues were distinguished. In groups III and IV, the surface area of both types of adipose tissue was significantly larger than that in group I. Despite the progressive increase in visceral adipose tissue with age, a decrease in insulin sensitivity was found only in women aged >60 years. In addition, the oldest women observed 28% lower glucose consumption compared to those in the other groups. The reason was reduced insulin action in women approximately 10–15 years after menopause [15].

The most characteristic disorder of carbohydrate metabolism during the ageing period is a gradual increase in the value of fasting and post-meal insulinisation. This suggests that ageing is a process accompanied by the development of tissue insulin resistance [16]. The impact of ageing on carbohydrate management is very complex. The interaction of many factors related to these processes may contribute to the deterioration of glucose tolerance. These include decreased physical activity, excessive body weight, the consumption of certain drugs, the co-occurrence of many diseases and age-related complex disorders of mechanisms controlling glycaemic homeostasis [11,17].

For this reason, several nutritional assessment systems such as GI, GL and glycaemic equivalents have been proposed. These measures are derived from the area under the glycaemic curve obtained at 2 h after the ingestion of the food product and the reference solution. Systematic studies have shown that both GI and GL are stronger predictors of postprandial glucose and insulin levels than carbohydrate content alone [18,19]. However, there is wide variability in the postprandial response within and between individuals.

The increased interest in the glycaemic index (GI) and glycaemic load (GL) of foods is due to the association of these indices with chronic diseases [13]. The values of the glycaemic index (GI) of products and foods, and thus the glycaemic response after consumption, vary. A number of factors influenced the rate of carbohydrate digestion and release of its enzymatic decomposition products. They are related to, among others, the degree to which the product is processed, the type of thermal treatment, the profile of carbohydrates contained in them, the structure of starch, the temperature of a meal and its consumption rate as well as the presence of other nutrients, such as proteins, fats and dietary fibre in addition to organic acids and anti-nutritive components [13,20,21,22,23,24,25,26,27].

Conditions closely related to disorders of carbohydrate metabolism are insulin resistance and diabetes mellitus. The results show that low-GI rations can improve the health of people with type 2 diabetes. Meals characterised by low GI values reduced post-prandial hyperglycaemia as well as insulin response among patients. A low-GI diet can also help restore tissue insulin sensitivity while reducing medication doses [28,29,30]. Knowledge of the dynamics of the postprandial glycaemic response is important in nutrition management, diabetes research, nutritional care of patients and self-management. In everyday life, the most important factor influencing the occurrence of hyperglycaemia is food intake. However, the large variability in the dynamics of glycaemic responses across foods, in addition to the differences between groups of individuals, necessitates the study of the glycaemic index (GI) of the same food in different groups of individuals, not only among people with varying BMIs, but also those with specific disease entities such as diabetes or cardiovascular disease. Studies determining the GI of foods and dishes have only been performed with healthy, young, normal weight subjects. What has not been studied is how specific foods affect postprandial glycaemia among other groups of people. Such measures can greatly facilitate the non-pharmacological treatment of, e.g., obesity or diabetes. This is especially important for diabetic patients who need to ensure tight glycaemic control to reduce diabetic complications. In the prevention of hyperglycaemia, information about the height and timing of the postprandial glucose peak in diabetic patients who depend on exogenous insulin to reduce postprandial glycaemia is important [31].

This information is also important for the large population of pre-diabetic individuals, as these patients may benefit from a lower glycaemic response profile to halt the development of diabetes [32]. In order to maintain control over the post-food reaction, knowledge of the metabolic effect of different foods in individuals is required. The predictor, which is often used in everyday life to predict postprandial glycaemia, is the carbohydrate content. However, there are large differences in the extent to which different sources of carbohydrates increase blood glucose levels: many studies have shown that the consumption of food containing equal portions of carbohydrate, and even the consumption of exactly the same food, produces distinctly different glycaemic responses [33,34,35].

In the scientific literature, studies demonstrating the effect of a low GI diet on selected anthropometric parameters and indicators of nutritional status of the body can be found. A study by Bai et al. [36] found that higher values of BMI and waist circumference were associated with an increased risk of type 2 diabetes among older people. However, data on the effect of nutritional status of the body on the glycaemic response after the consumption of specific foods and products are lacking.

Therefore, the main aim of this study was to evaluate the effect of the body mass index (BMI) values and body composition indices of women aged 50–80 years on glycaemic responses after the consumption of selected vegetarian dishes based on traditional and partially modified recipes. It should be noted that the study group consisted of women who had not been diagnosed with diabetes or other diseases that may affect carbohydrate metabolism. In this study, vegetarian dishes were used, which are characterised by their ease of preparation, low production costs and high organoleptic qualities. In addition, a diet that excludes meat products has health-promoting benefits such as reducing the risk of metabolic syndrome [37]. The novelty and value of the study is that it was carried out among older people with a different BMI and body composition. The results obtained indicate the need for, and may be the basis for, further research into predicting glycaemic response in different groups of people, taking into account their personal characteristics.

## 2. Materials and Methods

### 2.1. Characteristics of Persons Participating in the Study

Participants in the study were people who attended the University of the Third Century in Wroclaw. Before starting the recruitment, participants were presented with the objective and the next steps of the study. In case of doubt, any interested person could ask questions. The study was approved by the Bioethics Committee at the Medical University of Wrocław (KB-848/2021).

Participants wishing to participate in the studies had to meet the following criteria:

- Age of 50–80 years;
- Good health in self-assessment;
- No special diets and preference for varied dietary rations;
- No medication influencing carbohydrate metabolism;
- Moderate physical activity (no competitive sport);
- Non-smoker;
- Overnight 10 h fasting prior to the study;
- To agree in writing to participate in the study.

**Figure 1:**
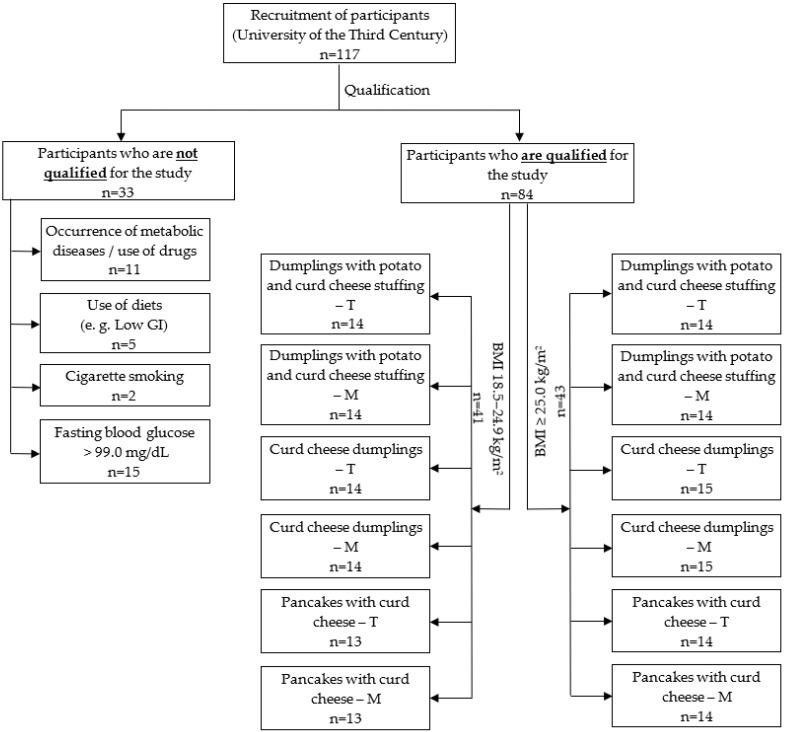
The scheme of the recruitment procedure. T—traditional version meals; M—modified version meals.

In order to check whether the volunteers met the criteria for entering the study, each person was asked to complete a questionnaire consisting of questions about their health status, medication use, physical activity, use of stimulants and eating habits (24 h interview repeated three times before the study). In the next stage, fasting glucose was measured with the Accu-Chek Softclix automatic lancetractor (Roche Diagnostics, Rotkreuz, Switzerland) and Accu-Chek Active glucose meter (Roche Diagnostics, Rotkreuz, Switzerland). Individuals who initially qualified for the study but whose fasting blood serum glucose levels exceeded 99.0 mg/dL were excluded from this study (15 individuals). Based on the obtained data, 33 subjects were excluded from this study. The study involved 84 women who were divided into two groups depending on their BMI value (group with BMI 18.5–24.9 kg/m^2^ and group with BMI ≥ 25 kg/m^2^). Although the study was conducted with human participants, the study group may appear small. Nevertheless, it follows the guidelines in the methodology and is considered reliable and commonly used in studies of the glycaemic indexes of products and foods [38,39]. A diagram of the recruitment process is shown in Figure 1.

Anthropometric measurements were taken to assess the nutritional status of the study participants. Body composition was analysed using the bioelectrical impedance method with an Accuniq BC380 medical device (Daejeon, Korea) with a built-in scale and equipped with an ultrasonic body height meter. Parameters such as height, body weight as well as fat mass, fat free mass, muscle mass, total body water, intracellular and extracellular water content were determined during the study.

This study was conducted under fasting conditions without shoes, top clothes, metal items, and after taking an upright posture. Body weight was measured on an empty stomach, with an accuracy of 0.01 kg, and the height was measured with an accuracy of 0.1 cm. In addition, on the basis of the obtained parameters, the BMI was calculated, expressing the quotient of body weight (kg) and square of body height (m^2^). Waist circumference (midway between the iliac crest and the terminal line of the ribs) and hip circumference (in the widest part at the level of the vertebral body) were measured with the use of anthropometric tape SECA (Germany; MDD 93/42 EEC certified EU standard for medical devices) with an accuracy of 0.1 cm, in a standing position. For each participant, the WHtR value (ratio of waist circumference to height—expressed in cm) and the WHR index (ratio of waist circumference to hip circumference—expressed in cm) for subjects with BMI ≥ 25.0 kg/m^2^ were calculated. The persons participating in the study were informed to continue their eating habits and physical activity during the study. The characteristics of the surveyed persons are presented in Table 1.

### 2.2. The Tested Meals

Vegetarian dishes—namely dumplings with potato and curd cheese stuffing, curd cheese dumplings and pancakes with curd cheese—were selected for this study. These are dishes that are very popular not only in Poland but also in other countries. These dishes are characterised by their high organoleptic qualities, low cost of production and ease of preparation, which is why they are popular not only among those who exclude meat from their diet. Dumplings with potato and curd cheese stuffing and curd cheese dumplings are mainly prepared as dinner dishes, while pancakes with cheese can be a breakfast dish or dessert. Therefore, the results of the present study can be used in nutrition planning of different societies.

Each dish was prepared in two variants: the first assumed the preparation of dishes according to a traditional recipe (T) using ingredients typical for the dish. The second assumed a partial modification of the composition by replacing plain wheat flour (type 471) with whole grain wheat flour (type 1630) (M). The other ingredients of the recipe were not changed. All the dishes were prepared independently in the catering laboratory of the Department of Human Nutrition at Wrocław University of Environmental and Life Sciences.

### 2.3. Nutritional Value of the Tested Meals

In order to assess the nutritional value of the tested dishes, each was prepared according to a specific recipe and then homogenised. In the samples prepared as such, the dry matter, ash content, energy value, protein, fat and dietary fibre content were determined. The content of carbohydrates assimilable in portions of the examined dishes was calculated by subtracting the content of ash, fat, protein and dietary fibre from the dry matter content. The energy value of the tested dishes was determined using the Rosenthal method [40]. The determination of water and dry matter content, total ash, protein, fat and dietary fibre was performed using standard Association of Official Analytical Chemists (AOAC) methods [41]. Each analysis was performed in triplicate. The nutritional value and weight of the served dishes containing 50 g of assimilable carbohydrates is shown in Table 2.

### 2.4. Method for Determining the GIs of Meals

Tests of GIs of food were performed according to ISO/FDIS 26642:2010 Food products—Determination of the glycaemic index (GI) and recommendation for food classification and according to the procedures recommended by FAO/WHO [38,39].

The day before the test, the test subjects were asked to reduce alcohol consumption, caffeinated drinks and intense physical activity. Each examination was preceded by a 10 h night fast.

The determination of the GI determination of the tested foods began with the determination of glycaemic curves after the consumption of a reference glucose solution consisting of 50 g of crystalline glucose dissolved in 250 cm^3^ of boiled water (the solution was served in transparent disposable containers). Glucose concentrations in capillary blood drawn from the finger were measured at fasting and then 15, 30, 45, 60, 90, and 120 min after the start of the consumption of the standard solution, which had to be consumed within 5–10 min. Blood glucose concentrations after the ingestion of the glucose solution were measured twice. The first measurement was taken at the beginning of the study and the second at the end of the experiment. The study was considered completed when the participant consumed the food prepared according to the traditional recipe (T) and the modified recipe (M).

The same was done for the tested dishes. GI marking of dishes took place in the morning, on weekdays. Volunteers were served a portion of a dish containing 50 g of assimilable carbohydrate and then blood glucose levels were determined at the same intervals as the glucose solution. The meals were prepared at the study site on the same day and served to the volunteers in white disposable dishes. Each dish had to be consumed within 10–15 min.

Each tested dish was consumed by a group of at least 13 people. It was also assumed that a person consuming a dish prepared according to a traditional recipe (T) must also consume a dish prepared according to a modified recipe (M). At the start of the study, each participant first consumed a dish prepared according to the traditional recipe (T) and then a modified dish (M). In the fasting subjects, capillary blood from the fingertip was collected using an Accu-Chek Softclix automatic puncture machine (Roche Diagnostics, Rotkreuz, Switzerland). After each blood drop collection, the blood glucose level was measured with the Accu-Chek Active glucose meter (Roche Diagnostics, Rotkreuz, Switzerland). The results were entered into a measurement sheet prepared for each participant and then entered into MS Excel 2013.

For each of the subjects, after the consumption of food and glucose, the areas under the glycaemic curves were graphically calculated by dividing them into triangles and trapeziums. Negative field values under glycaemic curves were not used for calculations. The food’s GI was determined by dividing the surface area under the curve obtained for the food tested by the surface area obtained for the glucose solution and multiplying the obtained result by 100.

### 2.5. Statistical Analysis

Statistical evaluation of the results obtained was carried out using STATISTICA version 13. 1 EN (StatSoft, Tulsa, OK, USA). The Shapiro–Wilk test was used to test the normal distribution of variables. In most cases, the data did not show normal distribution; therefore, the results were presented using the median and lower and upper quartiles. To test the materiality of differences in the results that were not characterised by normal distribution, a Box–Cox transformation was used. This transformation was to transform variables with diagonal distribution into variables with normal distribution. The Mann–Whitney U test was used to examine the differences in anthropometric parameters and body composition. To show statistically significant differences between the values of indices in the traditional and partially modified versions, the Student’s t-test for independent groups was used. Differences in GIs and glycaemic responses after the consumption of traditional and modified dishes depending on the BMI value were determined by one-factor analysis of variance (ANOVA) Tukey’s test for different N. The coefficient of variation (CV) values of glycaemia after 2-fold consumption of the reference solution did not exceed 19% (CV < 30% required). To assess the relationship between the GI of traditional and modified meals in groups of subjects with different BMI and anthropometric and body composition parameters, scatter plots were used.

## 3. Results

### 3.1. Glycaemic Responses after Consumption of the Reference Glucose Solution and the Tested Meals

**Figure 2:**
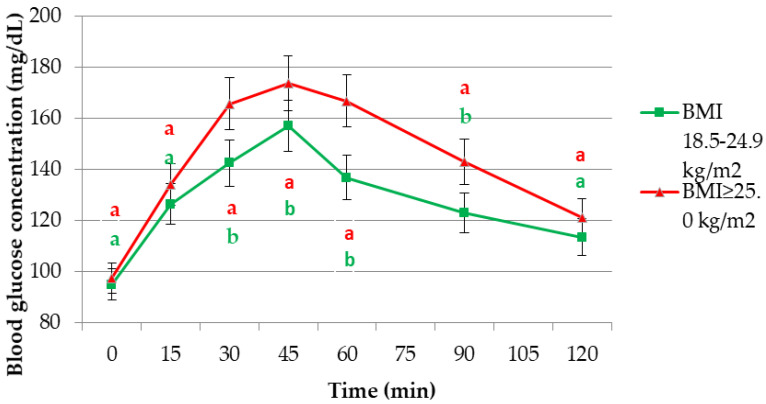
Curves of glycaemic response after the consumption of a standard glucose solution. **a**, **b**-statistically significant differences at *p* < 0.05.

After analysing the glycaemic responses after the consumption of the examined meals, differences were observed in the rate of increase and decrease in blood glucose concentration, as well as in its maximum concentration. After the consumption of the reference solution, the increase and decrease in blood glucose concentration was faster and more intensive than after the consumption of the examined dishes (Figure 2, Table 3, Table 4 and Table 5).

Figure 2 shows the glycaemic response curves after the ingestion of a standard glucose solution by women aged 50–80 years characterised by different BMI values. The highest blood glucose values during the 2 h test were observed in the group of women with BMI ≥ 25.0 kg/m^2^. Blood glucose concentrations in this group of women were also significantly higher at 30, 45, 60 and 90 min of the test in comparison to participants with correct BMI values.

Table 3 shows median glycaemic responses during the 2 h after the consumption of dumplings with potato and curd cheese stuffing, prepared in two recipe versions. The highest glycaemic response was observed at 45 min of the test, regardless of the version of the dish and the BMI of the participants. The modification of the recipe of the test dishes contributed to a lower glycaemic response after consumption. Glucose concentrations at 45, 60 and 120 min of the test, were significantly lower compared to the glycaemic response after the consumption of traditional dishes. This difference was observed, both in the group of women with a normal BMI and in the group with BMI ≥ 25.0 kg/m^2^. The modification of formulas contributed to a significant decrease in the field under the glycaemic curves in both groups of people (BMI 18.5–24.9 kg/m^2^: 2550.0 vs. 1590.0 j^2^ and BMI ≥ 25.0 kg/m^2^: 2898.7 vs. 2467.5 j^2^). Among women with BMI ≥ 25.0 kg/m^2^, the field under the glycaemic curve after the consumption of the modified product was significantly higher compared to the field obtained in the group of people with a normal BMI (*p* = 0.002).

Median blood glucose concentrations during the 2 h after consumption of curd cheese dumplings in the traditional and modified versions are shown in Table 4. The consumption of traditional curd cheese dumplings by the test participants resulted in the highest glucose concentration at 30 min of the test. The highest glycaemic response after the consumption of the modified product, in the group of women with normal BMI, was observed at 60 min of the test. Among overweight and obese participants, the highest glucose levels were recorded at 45 and 60 min of the test (127.0 mg/dL). Regardless of the participants’ nutritional status, the modification of the ingredients in the recipe of curd cheese dumplings contributed to lower glycaemic responses at 15, 30, 45 and 60 min of the test. In contrast, it was shown that a BMI ≥ 25.0 kg/m^2^ had a significant effect on glucose levels at 90 min of the test. This relationship was not observed in the group of women with normal BMI. Analysis of the data showed that BMI only significantly affected glucose concentrations at 90 min of the test and only for the modified product (*p* = 0.027). Modification of the product formulation, in each of the study groups, contributed to a reduction in the area under the glycaemic curves. It was also shown that, after consuming the traditional version of the dish, the field under the glycaemic curve was significantly higher among people with BMI ≥ 25.0 kg/m^2^ (*p* = 0.002).

Table 5 shows median blood glucose concentrations after the consumption of pancakes with curd cheese in the traditional and modified versions by the examined groups. In each of the study groups, the highest blood glucose levels after the consumption of pancakes with cheese in the traditional and modified versions were observed at 45 min of the study. Regardless of the BMI values of the study participants, the modification of the recipe of pancakes contributed to a significant reduction in the glycaemic response after their consumption (except for the measurement at 90 min) and a decrease in the area under the glycaemic curves. The blood glucose concentration in persons with BMI ≥ 25.0 kg/m^2^ after the consumption of the modified version of the dish was significantly higher at the 30th and 45th minutes of determination in comparison to those in women with normal BMI.

### 3.2. Values of GIs of the Tested Meals

Table 6 shows the GI values of dumplings with potatoes and curd cheese stuffing, curd cheese dumplings and pancakes with curd cheese consumed by women aged 50–80 years old with different BMIs. Based on the obtained results, it was observed that the modification of the traditional recipes of the studied dishes, involving the replacement of wheat flour type 471 with whole wheat flour type 1630, significantly influenced the lowering of their GIs, regardless of the BMI values of the study participants. The nutritional status of the women, in the case of dumplings with potatoes and curd cheese stuffing and curd cheese dumplings, was not a factor that significantly reduced the GI value of these dishes. A significant effect of the BMI on the GI value of the traditional and modified product was only observed for pancakes with curd cheese (*p* = 0.002 vs. 0.003, respectively).

**Figure 3:**
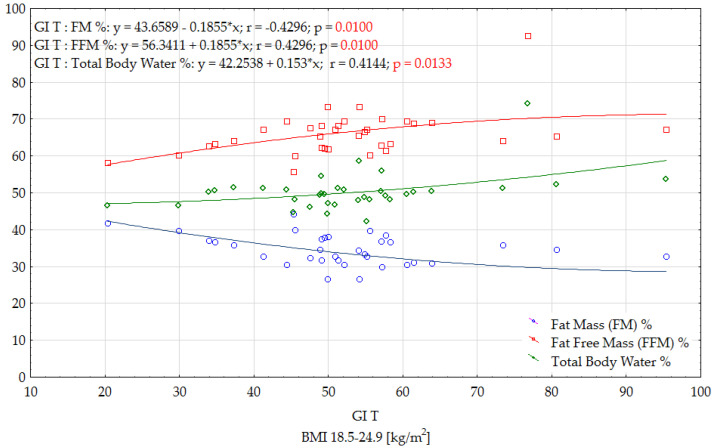
Effects of selected anthropometric parameters on the GI of traditional dishes in women with a BMI 18.5–24.9 (kg/m^2^).

**Figure 4:**
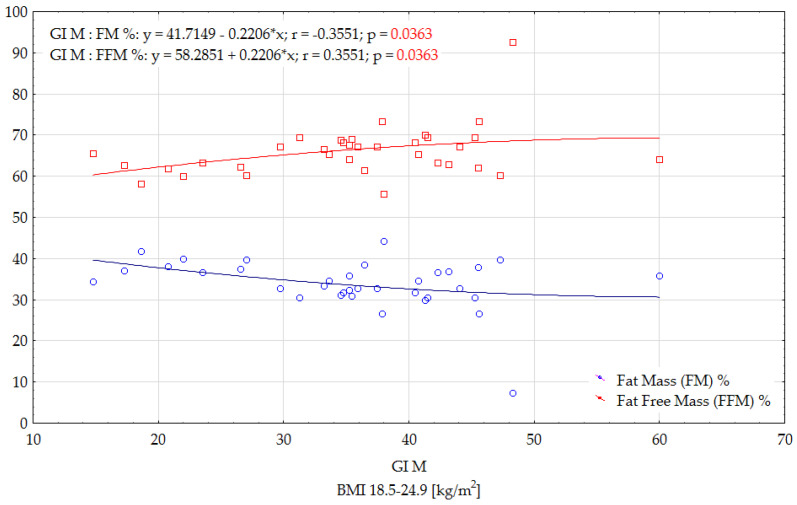
Effects of selected anthropometric parameters on the GI of modified dishes in women with a BMI 18.5–24.9 (kg/m^2^).

Figure 3 and Figure 4 show the influence of selected anthropometric indices that significantly affect the GI of the tested dishes, prepared according to traditional and modified recipes in a group of women with a BMI of 18.5–24.9 kg/m^2^. On the basis of an analysis of the obtained correlations, it was observed that the GI value of modified products depends on the percentage of body fat (FM%) and the percentage of fat free mass (FFM%), and, in the case of traditional products, also on the percentage of total body water (%). In the case of FM, a negative, moderate and significant correlation was observed. This means that higher GIs were observed in women with lower body fat. It was shown that the GI of the tested foods decreased with decreasing muscle tissue percentage and, in the case of traditional products, in addition to the decreasing percentage of total body water content.

**Figure 5:**
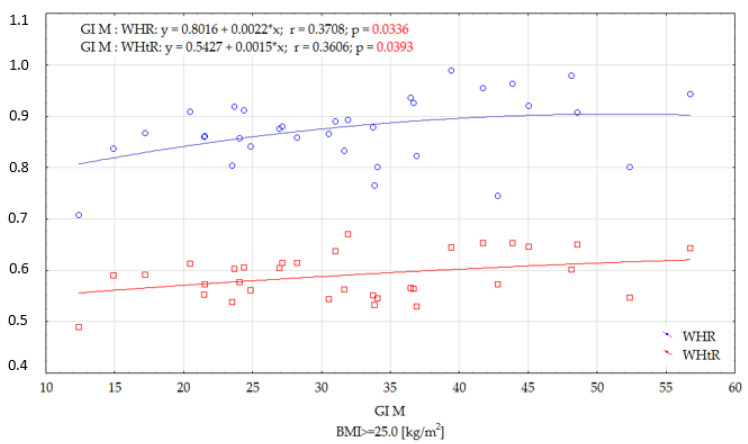
Effects of selected anthropometric parameters on the GI of modified dishes in women with a BMI ≥ 25.0 (kg/m^2^).

In the group of women with a BMI ≥ 25.0 kg/m^2^, significant correlations were only found between the GI of modified foods and the WHR and WHtR indices. These correlations were low, and positive, suggesting that the nutritional status of the women participating in the study significantly influenced the increase in GI of the tested foods (Figure 5).

## 4. Discussion

The modification of the recipes of the tested dishes contributed to significant changes in their nutritional value, especially in terms of dietary fibre content. It was also shown that the replacement of wheat flour type 471 with whole wheat flour type 1630 significantly reduced the GI values of dishes consumed by both women with BMI 18.5–24.9 kg/m^2^ and BMI ≥ 25.0 kg/m^2^. Glycaemic responses following food consumption in those with BMI ≥ 25.0 kg/m^2^ were higher compared to those with a normal BMI. However, the opposite trend was observed in the GI values of the tested foods. In the group of women with a normal BMI, the GI of the modified tested foods decreased with a decrease in the percentage of muscle tissue and, in the case of traditional foods, in addition to a decrease in the percentage of body water.

In the group of women with a BMI ≥ 25.0 kg/m^2^, only significant correlations were found between the GI of modified foods and the WHR and WHtR indices.

Research has been conducted in numerous scientific centres to determine the GIs of food. The GI values of marked food products and dishes were collected in the form of an international table [42]. In 2015, a scientific summit was held in Italy, where experts discussed the controversy over the usefulness of GI and GL. The panel of scientists confirmed the need to inform the public about GI and GL values, based on national guidelines and food composition tables [43].

To our knowledge, the GI studies of foods have primarily been conducted with young, healthy individuals with a BMI within the normal range. The available research results do not provide information on the glycaemic response after the consumption of specific foods by older people with varying BMI and body composition.

However, a growing body of scientific evidence suggests that glycaemic responses to the same foods vary considerably between individuals. In addition to the composition, preparation and properties of the consumed foods, individuals’ glycaemic responses vary according to the physiological and genetic variables as well as their microbiome [44,45]. While appreciating the benefits of being guided by the values of GIs when planning diets for many diseases, it is justified to continuously expand this base to include dishes popular in the nutrition of various societies. Additionally, taking into account differences in the carbohydrate economy of individual people, it seems advisable to carry out this type of research in different groups of people. In addition, food producers, researchers and consumers are all looking for new raw materials and methods to compose food recipes with high health-promoting qualities.

Based on scientific articles highlighting the fact that dietary fibre can contribute to lowering the GI of dishes [46,47,48], this study attempted to modify vegetarian dishes. Wheat flour type 471 was substituted for whole wheat flour type 1630, thereby significantly increasing the dietary fibre content of the tested dishes (Table 2). Similar treatments were also used by other researchers. Thondre and Henry [49] conducted an experiment in which the effect of the addition of 2, 4, 6 and 8 g of barley β-glucans to wheat flat cakes (this dish is prepared from unfermented dough and is a basic component in Indian cuisine) on their GI values was tested. It was observed that, as the proportion of β-glucans increased, the GI of the pancakes decreased. The value of the area under the glycaemic curve after consuming a portion of bread with 8 g of β-glucans was 47% lower than after consuming bread without added dietary fibre [49]. Similar relationships were shown in our study (Table 2). After the consumption of modified foods, glycaemic responses were lower compared to their traditional counterparts (Table 3, Table 4 and Table 5). Thus, it was observed that food modification had a significant effect on lowering the GI values of foods, both among those with a BMI of 18.5–24.9 and those with a BMI above 25.0 kg/m^2^ (Table 6). The beneficial effect of dietary fibre on GI reduction was also demonstrated in studies by Borczak et al. [47] and Naknaen et al. [48].

Dolna et al. conducted a study to determine the GI value of pancakes with curd cheese and curd cheese dumplings, among others. Only 14 young, healthy subjects (mean age 26.5 years) participated in this study. The GI values of these dishes were 35.0 and 12.6%, respectively [50]. The differences between the results of Dolna et al. and our own study are most likely due to different recipes and differences in metabolic responses between people of different ages. The ability to regulate blood glucose decreases with age. The decrease in glucose tolerance is explained by the influence of body fat and physical activity [51,52].

In a study conducted by Gabriely et al. in an animal model, it was observed that the amount of visceral fat increased with the progressive ageing of rats. The effect of insulin in ageing rats was significantly impaired. The increase in insulin resistance of fat tissue, liver and skeletal muscles has been markedly reduced by preventing the increase in body fat. Moreover, in experimental animals, a decrease in the glucose transporter GLUT4 concentration in skeletal muscles was observed, and it was considered to be one of the age-dependent causes of increased insulin resistance [53]. The above observations are identical to the results of our own research. In fact, it was shown that the glycaemic responses following food intake by those with a BMI ≥ 25.0 kg/m^2^ were higher compared to those with a BMI in the normal range. This is reflected in the values of the areas under the glycaemic curves—in each case, the areas among overweight/obese individuals are higher (Table 3, Table 4 and Table 5).

The progression of postprandial glycaemia and the GI value of a product primarily depends on its physical properties and chemical composition. However, a different glycaemic response can be observed in each person after eating the same food. This is due to variations in metabolic rate (e.g., according to age, weight and body composition). In one study, the body compositions of 220 healthy individuals aged 26–75 years (BMI 18.7–40.4 kg/m^2^) were analysed. Visceral adipose tissue content among those aged 26–44 years was significantly lower than in the group aged 60–75 years. In addition, older people had a lower subcutaneous adipose tissue content. The study confirmed that the amount of visceral adipose tissue increases with age, which significantly impairs tissue insulin sensitivity [54]. In our study, it was additionally shown that the WHR value is a factor influencing the GI values of foods—as the WHR value increased, a significant increase in the GI values of modified foods was observed (Figure 5). Higher WHR values are indicative of fat accumulation in the abdominal region. According to Imbeault et al., fat accumulation in the abdominal region has the greatest impact on impaired glucose tolerance. In addition, they found that fasting glucose levels and the area under the glycaemic curve after an oral glucose load were higher in patients aged 50–70 years [55]. In addition, a study by Sandra Hirsch et al. showed that there are inter-individual differences in glucose concentrations, even in healthy individuals. These may be caused by stress, changes in gastrointestinal motility and absorption rate and sleep deprivation, which reduces insulin sensitivity in healthy individuals. In addition, the composition of the meal consumed before the experiment or the degree of chewing can also affect glucose absorption [56,57].

The present study showed that the GI of meals consumed by those with BMI ≥ 25.0 kg/m^2^ was lower than that of food consumed by women with normal values of this index (Table 6). Only the modified version of dumplings with potatoes and curd cheese stuffing had a higher GI, but this difference was not statistically significant compared to the value obtained in the group of women with normal BMI (34.4% vs. 30.8%). The insignificant difference in GI values may be due to the recipe of this dish (low proportion of flour substituted). It was initially hypothesised that, in the group of women with a BMI > 25.0 kg/m^2^, the GI values of the tested dishes would be higher than the GIs determined with women of normal nutritional status. This could also be evidenced by positive and significant correlations between the GI values of the tested dishes in the modified version and the values of the WHR and WHtR indices (Figure 5). On the other hand, it was noted from the results that the areas under the glycaemic curves after the consumption of the tested dishes, both traditional and modified, are higher in the group of overweight and obese women. However, the GI values of these foods are lower than in the group of women with normal BMI. This is due to the fact that women with an abnormal body weight had a significantly higher glycaemic response after consuming the standard solution (Figure 2). This is a factor that, according to the GI formula, significantly affects the final GI value. Zakrzewski et al. conducted a study involving normal weight and obese girls. Its purpose was to compare, in these two groups of subjects, the glycaemic response after consuming high- and low-GI breakfast meals. Peak blood glucose levels were significantly higher after eating high-GI meals, but only in the group of obese girls (*p* = 0.023). Such relationships were not observed in the group of girls with normal body weight (*p* > 0.05). Additionally, the AUC after obese subjects consumed high-GI breakfasts was 13% greater than after consuming low-GI meals (*p* = 0.006). The area was only 4% greater in the normal weight group (*p* = 0.072) [58]. Additionally, in the present study, glucose levels after the consumption of tested foods were higher in the group of subjects with a BMI ≥ 25.0 kg/m^2^ (Table 3, Table 4 and Table 5). This relationship applied to both traditional and modified versions of dishes.

The weakness of the article is the lack of a group of people aged 50–80 years with a BMI < 18.5 kg/m^2^. When recruiting people for research, the authors were looking for people with low BMI, but no one with such parameters came forward. The lack of people with BMI < 18.5 kg/m^2^ was probably due to the fact that the people recruited for the study were students of the Wroclaw Universities of the Third Age. These were most often people who actively participated in various types of activities offered by universities, as well as in the life of their families. Research by Zielińska-Więczkowska et al. has shown that students of the Third Age universities are overwhelmingly satisfied with their lives. The quality of life of these people (age: 55–81 years) was stable and satisfactory [59]. Significant differences in the quality of life on a physical scale between the students of the Third Age universities (to their advantage) and the elderly who did not undertake such a form of activity were shown in the studies by Kozieł and Trafiałek [60]. Research also shows that older people who are satisfied with their lives are more likely to be overweight and obese than those who are in a negative mood. The second group of people is more often characterised as undernourished [61]. When planning the study, the authors did not take into account whether the women were peri- or postmenopausal. Biochemical markers of insulin resistance and inflammation were also not included in this study. This is a pilot study to see whether there are significant relationships between the selected parameters of nutritional status in older women and the GI values of the meals they eat. As body mass and body composition have been shown to significantly shape the GI values of meals, in the next stage, the authors plan a larger study involving both men and women, with blood biochemical tests and a detailed breakdown of the individuals into different BMI categories (with a breakdown into overweight groups and different degrees of obesity).

The results of the study can contribute to expanding the existing GI database of foods and dishes. Knowing the differences in the glycaemic response after the consumption of the same products by people with different BMI and nutritional status indices, the results of the study can be a starting point for the creation of tables with the GI values of foods and dishes for different groups of people.

## 5. Conclusions

According to the knowledge of the authors, the results of this work are the first to show the influence of the BMI values of elderly people on the glycaemic response and thus the GI values of vegetarian dishes. This study showed a beneficial effect of recipe modification, consisting of replacing wheat flour type 471 with wheat flour type 1630, on increasing the content of dietary fibre, which significantly shapes the glycaemic response. The unpredictability of individual glycaemic responses places limitations on the clinical utility of the GI. Accepting that glycaemic responses are indeed unpredictable and dependent on individual subjects would show a new perspective on the utility and limitations of the GI. This should encourage scientists to investigate the causes of the internal differences in AUC in order to refine the test procedures to minimise the differences. Overall, while GI is a valuable parameter, its use in the absence of knowledge of a person’s specific glycaemic response is imperfect. Due to the confirmed differences in the glycaemic responses of individuals after eating the same food, it is advisable and necessary to carry out similar studies in different groups of individuals. This may facilitate the non-pharmacological treatment of people with nutrition-dependent diseases and contribute to their prevention.

## Figures and Tables

**Table 1:** Characteristics of the persons examined (*n* = 84).

Parameter	BMI 18.5–24.9 kg/m^2^ (*n* = 41)		BMI ≥ 25.0 kg/m^2^ (*n* = 43)		*p*
	Median	Q1; Q3	Median	Q1; Q3	
Age (years)	65.0	63.0; 66.0	64.0	63.0; 67.0	0.518
Body weight (kg)	60.2	55.5; 63.3	74.9	71.2; 78.4	<0.001
Height (m)	1.61	1.55; 1.64	1.54	1.55; 1.60	0.070
Waist circumference (cm)	79.0	77.0; 80.0	92.0	89.0; 97.0	<0.001
Hip circumference (cm)	95.0	93.0; 98.0	107.0	102.0; 110.0	<0.001
WHR	-	-	0.88	0.84; 0.92	-
WHtR	0.49	0.47; 0.51	0.59	0.55; 0.62	<0.001
Fat mass (%)	34.4	31.2; 37.6	44.2	41.4; 45.9	<0.001
Fat free mass (%)	65.6	62.4; 68.8	55.8	54.1; 58.6	<0.001
Muscle mass (%)	40.0	35.7; 42.8	34.7	33.2; 37.9	0.006
Total body water (%)	49.9	48.0; 51.3	44.4	43.1; 46.7	<0.001
Extracellular water (%)	49.2	47.3; 51.5	49.2	46.5; 50.6	0.245
Intracellular water (%)	50.8	48.5; 52.7	50.8	49.4; 53.5	0.245

Values represent the median of the variables in each group and values for the 25th and 75th quartiles in a given group (Q1; Q3); red colour indicates statistically significant differences in the values of selected nutritional status indices in relation to BMI values (*p* < 0.05).

**Table 2:** Nutritional value and weight of the tested meals.

		Dumplings with Potato and Curd Cheese Stuffing	Dumplings with Potato and Curd Cheese Stuffing	Curd Cheese Dumplings	Curd Cheese Dumplings	Pancakes with Curd Cheese	Pancakes with Curd Cheese
		T	M	T	M	T	M
Fat	(g/100 g)	3.8	3.7	3.2	4.1	8.5	8.6
	*p*	0.005		0.002		0.006	
Protein	(g/100 g)	5.0	5.4	10.5	14.1	10.5	10.8
	*p*	0.002		<0.001		0.006	
Assimilated carbohydrates	(g/100 g)	19.9	18.1	24.3	25.8	24.3	22.6
	*p*	0.001		0.008		0.001	
Dietary fibre	(g/100 g)	1.0	2.4	0.8	2.9	0.8	2.5
	*p*	<0.001		<0.001		0.002	
Energy value	(g/100 g)	123.8	118.0	161.2	182.3	192.0	194.1
	*p*	0.018		0.020		0.017	
Served portion	(g)	264.0	322.1	215.0	217.0	215.0	246.0
	*p*	0.068		0.084		0.072	

T—traditional version meals; M—modified version meals; the fat, protein, fibre, and energy contents are the average of three repetitions; statistically significant differences in the value of selected nutritional value parameters for traditional and modified dishes are marked in red (*p* < 0.05).

**Table 3:** Blood glucose concentration (mg/dL) of the study participants within 2 h after the consumption of dumplings with potatoes and curd cheese stuffing prepared according to the traditional and partly modified recipes.

Time (min)	BMI 18.5–24.9 kg/m^2^			BMI ≥ 25.0 kg/m^2^			*p*-BMI T	*p*-BMI M
	T *n* = 14	M *n* = 14	*p*	T n = 14	M n = 14	*p*		
	Me (Q1; Q3)	Me (Q1; Q3)		Me (Q1; Q3)	Me (Q1; Q3)			
0	88.0 (84.5; 89.5)	88.5 (86.0; 90.5)	0.001	96.5 (90.0; 98.5)	95.5 (89.5; 99.0)	0.001	0.011	0.059
15	99.0 (94.5; 105.5)	90.5 (89.0; 100.5)	0.107	107.5 (102.5; 112.5)	102.5 (97.0; 105.0)	0.319	0.073	0.037
30	114.0 (109.5; 117.5)	109.0 (100.0; 114.5)	0.069	126.0 (121.0; 136.0)	121.5 (109.5; 127.0)	0.108	0.010	0.018
45	124.5 (116.5; 135.0)	112.5 (105.5; 123.5)	0.001	130.5 (121.5; 142.0)	129.5 (115.0; 135.5)	0.001	0.247	0.013
60	121.5 (111.0; 130.0)	112.0 (102.5; 128.0)	0.001	128.5 (118.5; 144.0)	124.0 (116.5; 131.5)	0.001	0.307	0.059
90	109.0 (100.5; 116.0)	109.0 (99.0; 118.5)	0.840	118.0 (111.0; 133.0)	112.5 (108.0; 123.0)	0.510	0.073	0.338
120	99.0 (95.0; 100.0)	97.5 (92.5; 103.5)	0.001	106.0 (100.0; 116.0)	104.5 (100.0; 112.0)	0.001	0.048	0.095
Area under the curve (j^2^)	2550.0 (1839.4; 3474.4)	1590.0 (1098.8; 2758.1)	0.001	2898.7 (2422.5; 3682.5)	2467.5 (1886.2; 3041.2)	0.047	0.075	0.002

Values represent the median of the variables in each group, values provided in brackets represent values for the 25th and 75th quartiles in a given group (Q1; Q3); T—traditional version meals; M—modified version meals; *p*-BMI T/*p-*BMI M—*p*-value of traditional/modified versions of meals after consumption by persons with different BMIs (*p* < 0.05). Statistically significant differences are marked in red.

**Table 4:** Blood glucose concentration (mg/dL) of the study participants within 2 h after the consumption of curd cheese dumplings prepared according to the traditional and partly modified recipes.

Time (min)	BMI 18.5–24.9 kg/m^2^			BMI ≥ 25.0 kg/m^2^			*p* BMI T	*p* BMI M
	T *n* = 14	M *n* = 14	*p*	T *n* = 15	M *n* = 15	*p*		
	Me (Q1; Q3)	Me (Q1; Q3)		Me (Q1; Q3)	Me (Q1; Q3)			
0	95.5 (94.0; 97.0)	97.0 (94.5; 98.0)	0.001	97.0 (94.0; 97.5)	98.0 (97.0; 99.0)	0.001	0.824	0.066
15	125.5 (112.5; 130.0)	104.0 (101.0; 107.5)	0.001	120.0 (114.0; 129.0)	105.0 (102.5; 110.5)	0.001	0.622	0.177
30	138.5 (129.0; 142.0)	116.5 (109.5; 118.5)	<0.001	139.5 (134.0; 144.5)	117.5 (113.0; 122.0)	<0.001	0.578	0.149
45	137.5 (125.5; 148.0)	121.5 (119.0; 125.0)	0.001	131.5 (127.5; 145.5)	127.0 (119.0; 127.5)	0.001	0.996	0.227
60	128.0 (118.0; 134.5)	123.0 (116.5; 130.5)	0.001	123.5 (119.5; 130.5)	127.0 (121.5; 132.0)	0.001	0.759	0.169
90	113.0 (103.0; 119.5)	114.5 (111.0; 121.5)	0.403	115.0 (110.0; 119.00	121.0 (115.0; 125.0)	0.095	0.644	0.089
120	102.0 (98.5; 109.5)	105.0 (101.5; 113.0)	0.413	100.0 (99.5; 105.5)	115.0 (108.0; 118.5)	<0.001	0.429	0.027
Area under the curve (j^2^)	2797.5 (2396.2; 3671.2)	2047.5 (1560.0; 2296.9)	0.001	3247.5 (2160.0; 3607.5)	2497.5 (2013.7; 2748.7)	0.002	0.002	0.082

Footnote as in Table 3.

**Table 5:** Blood glucose concentration (mg/dL) of the study participants within 2 h after the consumption of pancakes with curd cheese prepared according to the traditional and partly modified recipes.

Time (min)	BMI 18.5–24.9 kg/m^2^			BMI ≥ 25.0 kg/m^2^			*p* BMI T	*p* BMI M
	T *n* = 13	M *n* = 13	*p*	T *n* = 14	M *n* = 14	*p*		
	Me (Q1; Q3)	Me (Q1; Q3)		Me (Q1; Q3)	Me (Q1; Q3)			
0	95.0 (90.0; 97.0)	93.0 (88.0; 98.0)	0.715	95.0 (92.0; 98.0)	97.0 (96.0; 99.0)	0.076	0.443	0.024
15	110.0 (105.0; 120.0)	102.0 (93.0; 104.0)	0.003	110.0 (104.0; 130.0)	102.0 (100.0; 108.0)	0.003	0.403	0.170
30	125.0 (115.0; 135.0)	105.0 (103.0; 108.0)	0.003	130.0 (115.0; 140.0)	111.0 (109.0; 123.0)	0.003	0.337	0.019
45	129.0 (115.0; 132.0)	114.5 (109.0; 119.0)	0.004	134.0 (125.0; 138.0)	125.0 (118.0; 128.0)	0.044	0.068	0.017
60	119.0 (110.0; 121.0)	114.0 (110.0; 119.0)	0.003	124.0 (113.0; 134.0)	118.0 (110.0; 125.0)	0.003	0.215	0.441
90	110.0 (103.0; 115.0)	107.0 (97.0; 114.0)	0.338	110.0 (106.0; 123.0)	110.0 (105.0; 118.0)	0.455	0.222	0.163
120	101.0 (99.0; 106.0)	99.0 (91.0; 103.0)	0.003	102.0 (100.0; 111.0)	100.0 (100.0; 110.0)	0.003	0.274	0.061
Area under the curve (j^2^)	2122.5 (1972.5; 2827.5)	1462.5 (1185.0; 2403.7)	0.003	2625.0 (2032.5; 3116.2)	1672.5 (1147.5; 1935.0)	0.002	0.008	0.012

Footnote as in Table 3.

**Table 6:** Glycaemic indices of meals prepared according to the traditional and partly modified recipes.

Meal Type	BMI 18.5–24.9 kg/m^2^			BMI ≥ 25.0 kg/m^2^			*p* BMI T	*p* BMI M
	T	M	*p*	T	M	*p*		
Dumplings with potatoes and curd cheese stuffing	50.0 (47.1; 53.0) *n* = 14	30.8 (21.8; 39.2) *n* = 14	0.001	44.4 (32.0; 54.7) *n* = 14	34.4 (28.7; 43.3) *n* = 14	0.001	0.829	0.226
Curd cheese dumplings	54.9 (47.6; 69.3) *n* = 14	38.4 (36.1; 43.5) *n* = 14	0.001	44.8 (37.4; 52.3) *n* = 15	36.5 (32.9; 48.4) *n* = 15	0.001	0.063	0.964
Pancakes with curd cheese	55.5 (45.5; 57.7) *n* = 13	35.4 (23.5; 43.2) *n* = 13	0.003	35.1 (33.4; 40.4) *n* = 14	23.5 (17.2; 27.1) *n* = 14	0.003	0.002	0.003

Footnote as in Table 3.
